# 

**Published:** 1907-10-15

**Authors:** 


### CONTENTS

Event and Comment - - - - - - - - 485
Propagandism of Dental Education in Our Schools, *By George Zederbaum, D.D.S.* 487
The Schools and the Teeth - *By Dr. Charles O. Kimball, D.D.S.* 500
Artificial Enamel - - - - *By R. B. Tuller, D.D.S.* 512
Misconceptions Concerning Porcelain *By Louis Ladewich, D.D.S.* 519
The Inlay Matrix - - - - *By J. Q. Byram, D.D.S.* 522
A Gold Inlay - - - - *By Dayton D. Campbell, D.D.S.* 525
Indents - - - - - - - - - - - 527
Announcements - - - - - - - - - 537

